# Polydopamine-Based Surface Modification of ZnO Nanoparticles on Sericin/Polyvinyl Alcohol Composite Film for Antibacterial Application

**DOI:** 10.3390/molecules24030503

**Published:** 2019-01-30

**Authors:** Lisha Ai, Yejing Wang, Gang Tao, Ping Zhao, Ahmad Umar, Peng Wang, Huawei He

**Affiliations:** 1 State Key Laboratory of Silkworm Genome Biology, Biological Science Research Center, Southwest University, Chongqing 400715, China; als123@email.swu.edu.cn (L.A.); taogang@email.swu.edu.cn (G.T.); zhaop@swu.edu.cn (P.Z.); 2 College of Biotechnology, Southwest University, Chongqing 400715, China; modelsums@email.swu.edu.cn; 3 Chongqing Key Laboratory of Sericultural Science, Chongqing Engineering and Technology Research Center for Novel Silk Materials, Southwest University, Chongqing 400715, China; 4 Department of Chemistry, College of Science and Arts and Promising Centre for Sensors and Electronics Devices, Najran University, P.O. Box: 1988, Najran 11001, Saudi Arabia; umahmad@nu.edu.sa

**Keywords:** ZnO nanoparticles, polydopamine, sericin, mechanical performance, antibacterial activity

## Abstract

Silk sericin (SS) is a type of natural macromolecular protein with excellent hydrophilicity, biocompatibility and biodegradability, but also has very poor mechanical properties. To develop sericin-based wound dressings, we utilized polyvinyl alcohol (PVA) to reinforce the mechanical property of sericin by blending PVA and sericin, then modified zinc oxide nanoparticles (ZnO NPs) on SS/PVA film with the assistance of polydopamine (PDA) to endow SS/PVA film with antibacterial activity. Scanning electron microscopy, energy dispersive spectroscopy and X-ray powder diffraction demonstrated ZnO NPs were well grafted on PDA-SS/PVA film. Fourier transform infrared spectra suggested PDA coating and ZnONPs modification did not alter the structure of sericin and PVA. Water contact angle and swelling tests indicated the excellent hydrophilicity and swellability of ZnO NPs-PDA-SS/PVA composite film. Mass loss analysis showed ZnO NPs-PDA-SS/PVA film had excellent stability. The mechanical performance test suggested the improved tensile strength and elongation at break could meet the requirement of ZnO NPs-PDA-SS/PVA film in biomaterial applications. The antibacterial assay suggested the prepared ZnO NPs-PDA-SS/PVA composite film had a degree of antimicrobial activity against *Escherichia coli* and *Staphylococcus aureus*. The excellent hydrophilicity, swellability, stability, mechanical property and antibacterial activity greatly promote the possibility of ZnO NPs-PDA-SS/PVA composite film in antibacterial biomaterials application.

## 1. Introduction

Wound dressing has a variety of functions, of which the first is to prevent continued bleeding through a certain degree of mechanical compression. In addition, wound dressing should have a certain degree of water absorption ability. It is also necessary for wound dressing to absorb the wound exudate in a timely manner, and at the same time to adequately maintain a moist environment to promote wound healing and reduce scarring formation [1]. Finally, some antibacterial properties are needed to prevent wound infection [2,3]. To meet the requirement of novel wound dressing, it is necessary to find natural materials with good hydrophilicity and biocompatibility that could be alternative candidates to traditional wound dressing. Natural silk produced by silkworm is composed of two parts: the outer part of silk collagen composition, known as sericin, and the inner part of core glial protein, known as silk fibroin [4,5]. Sericin is well known for having skin moisturizing properties as it has lots of amino acids with strong polar groups [6,7]. Sericin has huge potential in biomaterial applications due to its admirable hydrophilicity, biocompatibility and biodegradability [7]. More than 50,000 tons of sericin are discarded in waste water each year worldwide [8], causing an enormous waste of natural resources and environmental pollution. Hence, developing sericin-based biomaterials not only save resources, but also protect our environment. Heat treatment of silkworm cocoons at ambient or increased pressure is a preferred method of extracting sericin without introducing impurities [9,10]. Sericin is fragile in a relatively dry environment for its high content of random structures [11,12]. However, a certain degree of mechanical strength is required for a biomaterial. Therefore, reinforcing the mechanical properties of sericin is a huge challenge that must be resolved in expanding the application of sericin in biomaterials.

Recently, polyvinyl alcohol (PVA) has been extensively applied in biomaterials for its various excellent properties such as biocompatibility, hydrophilicity and mechanical performance [13]. PVA could effectively improve the mechanical properties of sericin and does not alter the other excellent properties of sericin [14,15]. In real life, the exposed skin wounds are susceptible to bacterial infections. Bacteria can enter into the deeper skin layers through wounds [16]. Therefore, it is required for sericin/PVA (SS/PVA) film to have the ability of preventing bacterial infection. Zinc oxide (ZnO) is a compound generally recognized as safe by the Food and Drug Administration of the United States of America (21CFR182.8991). ZnO has anti-viral, anti-bacterial and anti-fungal activities and minimal toxicity to humans [17,18,19,20]. ZnO nanoparticles (ZnO NPs) have a pronounced antibacterial ability with a high specific surface area to volume ratio. ZnO NPs can inhibit the growth of both Gram positive and Gram negative bacteria. The antibacterial activity of ZnO NPs is related to nanoparticles size. The smaller the size is, the greater the possibility for nanoparticles to have contact with the bacterial surface area. With the decrease of the particle size of ZnO NPs, its antibacterial properties increase. In addition, ZnO NPs have anti-corrosive and UV filtering properties. ZnO NP is easy to synthesize and has a type of green nanomaterials that possess good biocompatibility and biodegradability [21,22,23]. ZnO NPs have wide applications in food packaging and the cosmetics industry [22]. Hence, it is a good candidate to endow sericin with antibacterial activity for wound dressing. However, it is very difficult to graft ZnO NPs directly on the SS/PVA film.

Dopamine is an important neurohormone in the brain [24]. It has the catechins and amino groups with high affinity for metal ions, which can be used both as an ideal molecule covalent modification of starting point and a transition metal ions load anchor. These functional groups can pass on the basic properties of metal ions under the condition of having a strong restorative ability to further realize the emergence of a variety of mixed material [25]. With these benefits, polydopamine (PDA) is widely used in chemistry, biology, medicine, materials science and other fields. This performance promotes the deposition of metal on material surface [26]. PDA has excellent biocompatibility, biodegradability and hydrophilicity [27]. Taking advantage of these features, PDA could facilitate ZnO NPs being deposited on the SS/PVA composite film to endow it with antibacterial properties.

In this work, we first prepared SS/PVA film by blending sericin with PVA, soaked the composite film into PDA solution to coat PDA on its surface, and then immersed PDA-SS/PVA film into ZnO NPs solution to yield ZnO NPs-PDA-SS/PVA composite film. PDA was used to facilitate ZnO NPs being deposited on the SS/PVA composite film. Multiple techniques were performed by characterizing the composite films using scanning electron microscopy (SEM), Fourier transform infrared spectroscopy (FT-IR), X-ray diffractometry (XRD) and energy dispersive spectroscopy (EDS) to validate properties such as morphology and structure and also to prove the successful preparation of ZnO NPs on the film. Antimicrobial assays were carried out to investigate the antimicrobial activity of the composite film against *Escherichia coli* (*E. coli*) and *Staphylococcus aureus* (*S. aureus*).

## 2. Results and Discussion

**Figure 1:**
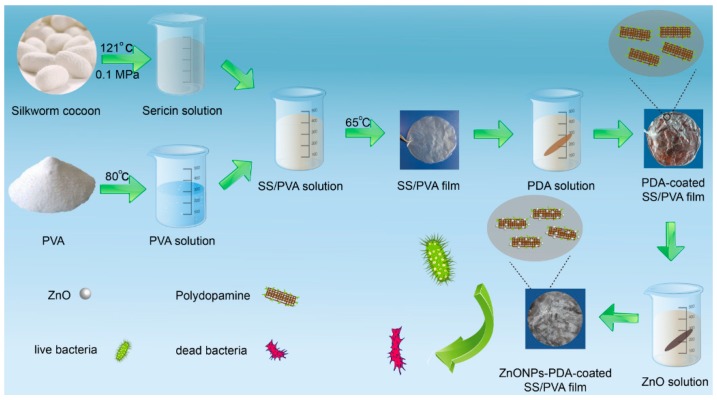
Schematic diagram of the preparation and antibacterial action of ZnO NPs-PDA-SS/PVA film.

ZnO NPs-PDA-SS/PVA composite film was prepared through the blending of sericin and PVA solution followed by PDA coating and ZnO NPs modification. The antibacterial activity of the composite film was tested to assess its usability in antibacterial applications such as wound dressing. The schematic diagram was shown in Figure 1.

### 2.1. SEM, EDS and XRD

**Figure 2:**
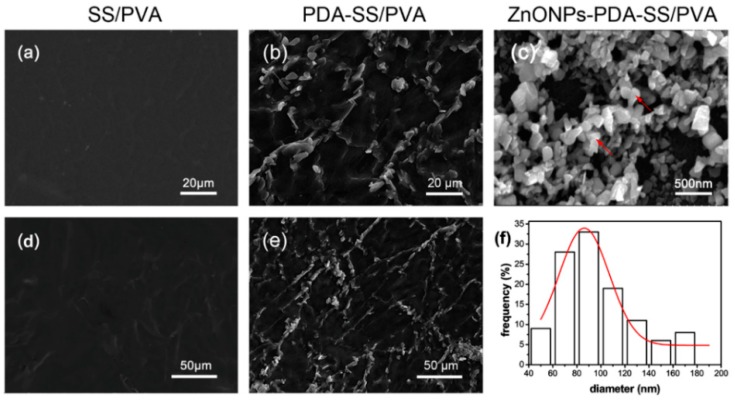
SEM of SS/PVA (**a**,**d**), PDA-SS/PVA (**b**,**e**) and ZnO NPs-PDA-SS/PVA films (**c**), respectively. The size distribution of ZnO NPs on the PDA-SS/PVA film (**f**).

SEM micrograph showed the surface morphology of the films. Figure 2a,d showed that SS/PVA film had a smooth surface, indicating the uniform blending of sericin and PVA. Figure 2b,e showed the surface morphology of PDA-SS/PVA film with a rough surface. PDA adsorption on the film is determined by monomer concentration, reaction time and temperature [14]. We carried out the experiment by constant stirring at 37 °C for 12 h in 2.0 mg/mL dopamine. PDA enhanced the interaction of sericin with ZnO NPs as a mediating agent. Figure 2c showed the surface morphology of ZnO NPs-PDA-SS/PVA composite film. The red arrows indicated ZnO NPs on the surface of PDA-SS/PVA film. Figure 2f showed the size distribution of ZnO NPs on the PDA-SS/PVA film. Most of ZnO NPs were in the size range of 60–120 nm, which implied its potential antibacterial activity.

**Figure 3:**
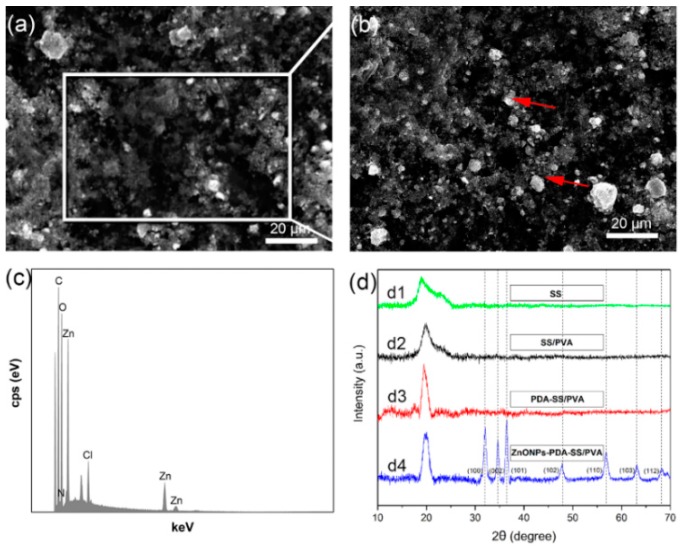
SEM characterization of ZnO NPs-PDA-SS/PVA film. (**a**,**b**) Field emission scanning electron microscope. ZnO NPs is indicated in red arrows. (**c**) A representative EDS spectrum of ZnO NPs-PDA-SS/PVA film. (**d**) The XRD profiles of sericin, SS/PVA, PDA-SS/PVA and ZnO NPs-PDA-SS/PVA films (d1–d4).

To verify whether ZnO NPs were well grafted on the film, we performed energy-dispersive spectroscopy (EDS) analysis. The EDS spectrum of the selected area of the composite film (Figure 3a) showed the well-defined peaks of Zn and O, confirming the existence of ZnO on PDA-SS/PVA film (Figure 3c). This result proved that ZnO NPs were well grafted on the composite film. We also noticed variant shape and size of ZnO NPs on the surface of the film (Figure 3b).

XRD showed sericin has an abroad and shallow diffraction peak at around 2θ = 19.2°. A broad peak located at 2θ = 19.8° indicated the semicrystalline structure of sericin (Figure 3d). No typical diffraction peak of PDA was observed, indicating that PDA coating did not affect the crystal structure of SS/PVA composite film. The diffraction peaks observed at 2θ = 31.3°, 34.0°, 36.2°, 47.5°, 56,6°, 63.0°, and 68.9° corresponded to the crystal planes (100), (002), (101), (102), (110), (103), and (112), indicating the hexagonal formation of Wurtzite structure in ZnO nanoparticles [28].

### 2.2. FT-IR

**Figure 4:**
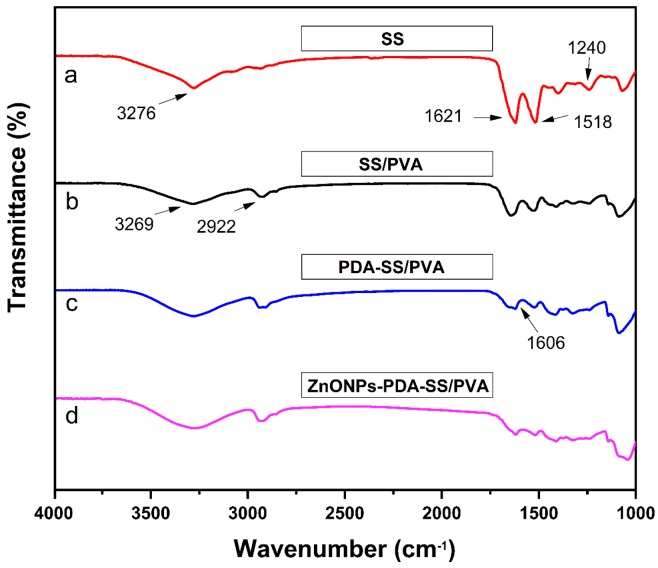
FT-IR spectra of the as prepared sericin and composite films (**a**–**d**).

FT-IR was performed to characterize the chemical composition of sericin, SS/PVA, PDA-SS/PVA, ZnO NPs-PDA-SS/PVA films, as shown in Figure 4. The amide I, II and III bands of sericin existed in all spectra, corresponding to the characteristic peaks located at 1621 cm^−1^, 1518 cm^−1^, and 1240 cm^−1^ [29]. PVA has characteristic O-H stretching vibration peaks at 3276 cm^−1^ (Figure 4a) and 3269 cm^−1^ (Figure 4b) [30]. The peak of 1606 cm^−1^ indicates the C=C stretching and N-H deformation vibration of indoles or indoline structures in PDA molecule [31], suggesting that PDA was successfully grafted on the surface of SS/PVA composite film. The FT-IR spectrum of ZnO NPs-PDA-SS/PVA film was similar to that of PDA-SS/PVA film, suggesting ZnO NPs modification does not affect the amide peaks of sericin and the characteristic peaks of PVA and PDA.

### 2.3. Wettability and Swellability

**Figure 5:**
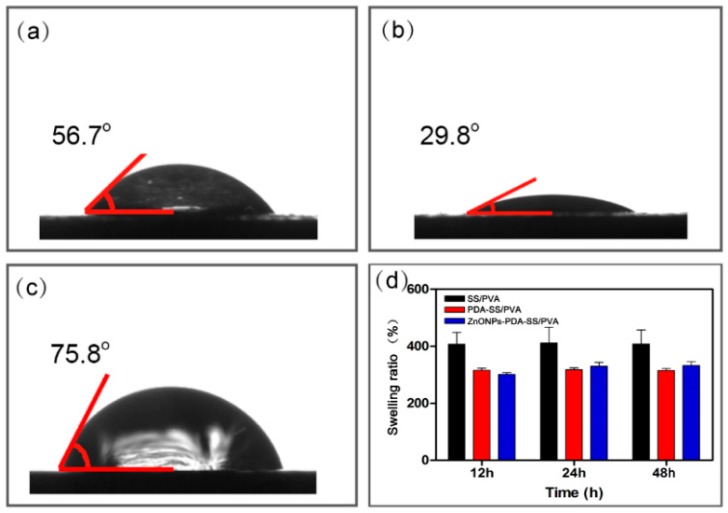
Water contact angle and swelling property of SS/PVA (**a**), PDA-SS/PVA (**b**), and ZnO NPs-PDA-SS/PVA films (**c**). Swelling ratio of these films (**d**) (*n* = 3 per group).

Figure 5a–c showed the instantaneous water-absorption of SS/PVA, PDA-SS/PVA and ZnO NPs-PDA-SS/PVA composite films. The water contact angle of PDA-SS/PVA film was 29.8°, which was the smallest among all of the tested films, indicating that it has the best water absorption ability. PDA is an important factor to affect water absorption as it has excellent hydrophilicity [25]. The water contact angle of SS/PVA film was 56.7°, indicating the film is hydrophilic. After ZnO NPs modification, the water contact angle increased to 75.8°, but it was still hydrophilic. ZnO NPs modification covered the hydrophilic groups on the surface of PDA-SS/PVA film, which thus resulted in the decrease of water absorption property.

The swellability of SS/PVA, PDA-SS/PVA and ZnO NPs-PDA-SS/PVA composite films is shown in Figure 5d. All the composite films with a dimension of 1 cm × 1 cm (length × width) were immersed into PBS buffer (pH 7.4) for 12 h, 24 h and 48 h, respectively. The swelling ratio of SS/PVA increased about 4 times, indicating SS/PVA film has an excellent swellability. The swelling ratio of PDA-SS/PVA and ZnO NPs-PDA-SS/PVA films were about 3 times of the control, suggesting these films have excellent swellability. The swelling ratio did not change over time. This may be explained by the fact that the sites of water molecule binding have been completely saturated in a short time. The excellent hydrophilicity and swellability indicated the potential of ZnO NPs-PDA-SS/PVA in wound dressing applications.

### 2.4. Mechanical Property

**Figure 6:**
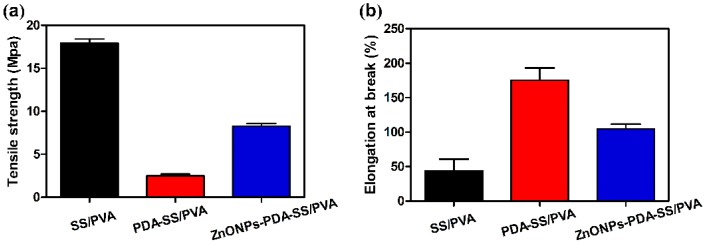
Mechanical properties of the films: (**a**) tensile strength and (**b**) elongation at break (*n* = 3 per group).

The mechanical properties of SS/PVA, PDA-SS/PVA and ZnO NPs-PDA-SS/PVA films were shown in Figure 6. SS/PVA had the highest tensile strength among all of the tested films, while PDA-SS/PVA film was the lowest. ZnO-PDA-SS/PVA film had a tensile strength of about 8 MPa, which is suitable for application of wound dressings. The elongation at break is an indicator of the material’s flexibility [32]. The results showed that PDA coating resulted in the increase of the elongation at break of SS/PVA film, while ZnO NPs modification reduced the elongation at break of PDA/PVA film (Figure 6b). PDA may interact with sericin/PVA through its amide and hydroxyl groups to form an extensive molecular network, and thus improved the elongation at break of SS/PVA film and decreased the tensile strength of SS/PVA film. ZnO NPs likely disrupted the molecular interaction between PDA and sericin/PVA partially, which resulted in the decrease of the elongation at break and the increase of the tensile strength. The elongation at break of these films ranged from 50% to 160%. The data showed ZnO NPs-PDA-SS/PVA film meets the requirements of wound dressing materials.

### 2.5. Mass Loss Analysis

**Figure 7:**
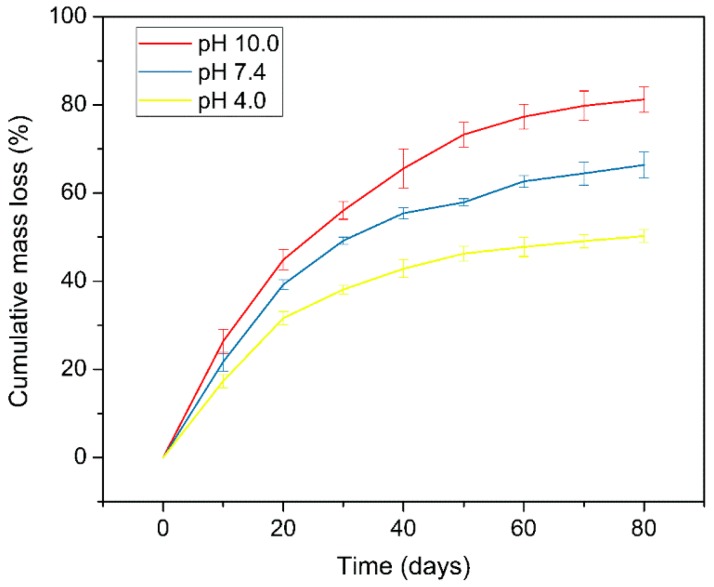
Mass loss of ZnO NPs-PDA-SS/PVA films under different pH conditions.

Sustained stability is one of the characteristics that a wound dressing is supposed to have. Here, we analyzed the mass loss of ZnO NPs-PDA-SS/PVA film under pH 4.0, 7.4 and 10.0 conditions to assess its stability. The cumulative mass loss of the composite film increased over time. Under pH 10.0, the mass loss of the film occurred faster than that under pH 4.0 and 7.4 conditions (Figure 7). This may be that sericin contains a number of acidic amino acids and has an isoelectric point of 3.8 [33]. While in an alkaline environment, sericin more easily reacts and can be more easily hydrolyzed [34]. The result showed that ZnO NPs-PDA-SS/PVA composite film has good stability.

### 2.6. Antibacterial Property

**Figure 8:**
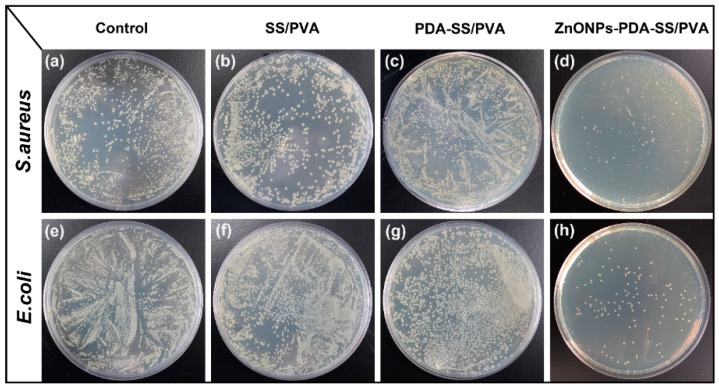
Antibacterial activity analysis of SS/PVA, PDA-SS/PVA and ZnO NPs-PDA-SS/PVA films against *E. coli* (**a**–**d**) and *S. aureus* (**e**–**h**).

The antibacterial property of ZnO NPs-PDA-SS/PVA, PDA-SS/PVA and SS/PVA films were analyzed against Gram-negative bacteria (*E. coli*) and Gram-positive bacteria (*S. aureus*), respectively. As shown in Figure 8, while compared to the control, the colonies number did not show a significant difference in the presence of SS/PVA and PDA-SS/PVA films. However, in the presence of ZnO NPs-PDA-SS/PVA film, the colonies number was much less than that of the control, indicating the good antibacterial property of the composite film against *E. coli* and *S. aureus*. The antibacterial activity of ZnONPs comes from three aspects: (a) Reactive oxygen species are produced from ZnO NPs, which destroy cell membrane, thus causing leakage of cytoplasmic contents, DNA damage, and cell death [35]; (b) Zinc ions from ZnO NPs can penetrate cell membrane to inhibit bacterial metabolic activity [32,36]; (c) The intracellular accumulation of ZnO NPs can destroy the cell wall of bacteria and affect DNA replication, leading to the death of bacteria [18].

### 2.7. Bacterial Growth Assay

**Figure 9:**
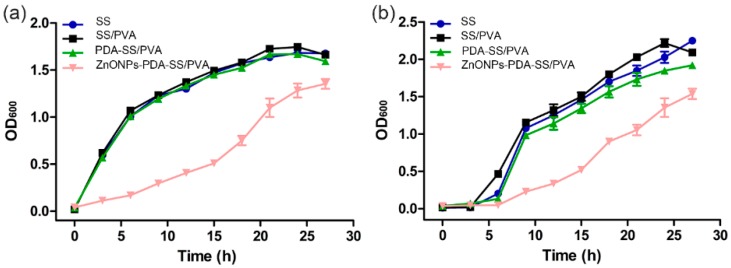
Bacterial growth curves of *E. coli* (**a**) and *S. aureus* (**b**) in the presence of different films.

To further confirm the bacteriostasis of ZnO NPs-PDA-SS/PVA film, the bacterial growth curve in the presence of the composite film was presented by measuring bacterial OD_600_, as shown in Figure 9. The bacterial growth profile was very similar between SS/PVA, PDA-SS/PVA films and the control. However, the bacterial growth was significantly retarded in the presence of ZnO NPs-PDA-SS/PVA film, suggesting ZnO NPs-PDA-SS/PVA film has a certain inhibitory effect on the growth of *E. coli* and *S. aureus*. This result was consistent with that of the colony counting method.

## 3. Materials and Methods

### 3.1. Materials

Silkworm cocoons were supplied by the State Key Laboratory of Silkworm Genome Biology, Southwest University, Chongqing, China. PVA and dopamine hydrochloride were purchased from Aladdin Corp (Shanghai, China). ZnO NPs (99.9%) was purchased from Guohua reagent (Shanghai, China). All other chemicals were of analytical grade and were directly used.

### 3.2. Fabrication of ZnO NPs-PDA-SS/PVA Film

Sericin was obtained from silkworm cocoons by means of autoclave at 121 °C for 30 min [37,38]. SS/PVA film was prepared as per our previous procedure [37,38]. Briefly, sericin solution (4%, *w*/*t*) and PVA solution (5%, *w*/*t*) were well mixed, and then dried at 65 °C in the Petri dishes to become SS/PVA film. SS/PVA film was immersed directly into fresh dopamine solutions (2.0 mg/mL, pH 8.5) at 37 °C for 12 h with continuous stirring. Then the PDA coated SS/PVA film was taken out and washed with MilliQ water to remove extra PDA. Furthermore, PDA-SS/PVA film was soaked in 61.7 mM ZnO NPs at 25 °C for 12 h. Finally, the ZnO NPs-PDA-SS/PVA composite film was produced after repetitive washing and was dried at 25 °C.

### 3.3. SEM, XRD, FT-IR and Mechanical Test

The surface morphology of ZnO NPs-PDA-SS/PVA, PDA-SS/PVA and SS/PVA films were imaged on a JSM-5610LV scanning electron microscopy (Tokyo, Japan) with a working voltage of 25 kV. The crystalline structure of the composite film was examined by PANalytical X’Pert, a powder X-ray diffraction system (Almelo, The Netherlands) over Bragg angles ranging from 10° to 80°. FT-IR spectra of the composite film were characterized on Thermo-Fisher Nicolet iz10 IR microscope (Framingham, MA, USA) over the wavenumber of 4000–400 cm^−1^.

SS/PVA, PDA-SS/PVA, ZnO NPs-PDA-SS/PVA films were prepared with a dimension of 4 cm × 1 cm (length × width). These films’ tensile properties were tested on a universal AG-X-plus testing machine (Shimadzu, Kyoto, Japan) equipped with a 1000 N loading cell. The crosshead speed was 5 mm/min. The thickness of the film was measured by SEM. To reveal the stress-strain relation, the recorded data was transformed into real stress (σ) and strain (ε) [39].

### 3.4. Hydrophilicity and Swellability

The hydrophilicity of ZnO NPs-PDA-SS/PVA, PDA-SS/PVA and SS/PVA were analyzed at 25 °C on a Krüss DSA100 system (Hamburg, Germany) via sessile drop contact angle. At five different positions, the process of water absorption was recorded by dispensing the water droplet on the surface of the sample.

The swelling of the composite films in phosphate buffer (PBS, pH 7.4) was measured by gravimetric method with a minor modification. The original films were weighed as *W*_1_. Then, the swelling films were immersed into phosphate buffer at 37 °C, then taken out at separate time intervals and gently removed by filter paper. The mass of swollen composite films was marked as *W*_2_. The swelling ratio (*S*) was calculated by the following equation:

*S* (%) = (*W*_2_ − *W*_1_) × 100%/*W*_1_ (1)

The same operation was performed at least three times under the same condition. The swellability of the film was presented by average data.

### 3.5. Mass Loss Analysis

A mass losing ratio analysis was conducted to analyze the stability of the composite films. First, the films were prepared with a dimension of 3 cm × 3 cm (length × width), and then soaked into PBS with different values (pH 4.0, 7.4, 10.0) at 25 °C, respectively. At given time intervals, the films were carefully taken out, washed, and weighed. The mass of the film before and after the treatment were recorded as *W*_3_ and *W*_4_, respectively. The test was repeated five times. The mass losing ratio (R) was calculated using the following equation:

R (%) = (*W*_3_ − *W*_4_) ×100%/*W*_3_ (2)

### 3.6. Antibacterial Test

The antibacterial activity of the composite film was assessed against gram-positive bacteria *S. aureus* and gram-negative bacteria *E. coli* as Pal’s protocol [40]. First, the bacteria were cultured in 10 mL Luria-Bertani (LB) medium (pH 7.4) under a constant vibrating velocity of 2.8× *g* at 37 °C overnight. Then the bacteria suspension was diluted with LB medium to an optical density at 600 nm (OD_600_) of 0.02, which was used as the control. The diluted bacteria suspension was cultured at 37 °C for 30 h in the presence of sterile ZnO NPs-PDA-SS/PVA, PDA-SS/PVA, SS/PVA films (1 cm × 1 cm), respectively.

The bacteria were cultured in 12-well plate in the presence of sterile composite films at 37 °C, and were collected at different time intervals to measure OD_600_. After 3 h, the bacterial suspension of each experiment was diluted for several times and then cultured on LB plate at 37 °C overnight. The antibacterial activity of the film was evaluated by counting the number of colonies on the plate. The assay was performed in triplicate for each independent experiment.

## 4. Conclusions

In this work, we prepared SS/PVA film with enhanced mechanical performance through blending sericin and PVA, and then grafted ZnO NPs on SS/PVA film via PDA to yield ZnO NPs-PDA-SS/PVA composite film. SEM confirmed the spherical shape ZnO NPs on PDA-SS/PVA film. The elemental composition and chemical composition of ZnO NPs-PDA-SS/PVA film were confirmed by EDS and FT-IR. The crystal planes of ZnO NPs were determined by XRD. Water contact angle and swelling analysis indicated the excellent hydrophilicity and swellability of ZnO NPs-PDA-SS/PVA film. The mechanical test validated the improved mechanical performance of the composite film. Mass loss analysis showed good stability of ZnO NPs-PDA-SS/PVA film under different pH conditions. ZnO NPs-PDA-SS/PVA film exhibited a certain degree of antibacterial effect against *E. coli* and *S. aureus*. The improved mechanical performance and antibacterial activity will greatly promote the application of ZnO NPs-PDA-SS/PVA composite film in antimicrobial biomaterials such as wound dressing.

## References

[1] Pan H., Fan D., Cao W., Zhu C., Duan Z., Fu R., Li X., Ma X. (2017). Preparation and Characterization of Breathable Hemostatic Hydrogel Dressings and Determination of Their Effects on Full-Thickness Defects. Polymers.

[2] Al-Omair M. (2015). Synthesis of Antibacterial Silver–Poly(ε-caprolactone)-Methacrylic Acid Graft Copolymer Nanofibers and Their Evaluation as Potential Wound Dressing. Polymers.

[3] Khil M.S., Cha D.I., Kim H.Y., Kim I.S., Bhattarai N. (2003). Electrospun nanofibrous polyurethane membrane as wound dressing. J. Biomed. Mater. Res. B.

[4] Kato N., Sato S., Yamanaka A., Yamada H., FUWA N., NOMURA M. (1998). Silk protein, sericin, inhibits lipid peroxidation and tyrosinase activity. Biosci. Biotechnol. Biochem..

[5] He H., Tao G., Wang Y., Cai R., Guo P., Chen L., Zuo H., Zhao P., Xia Q. (2017). In situ green synthesis and characterization of sericin-silver nanoparticle composite with effective antibacterial activity and good biocompatibility. Mater. Sci. Eng. C.

[6] Kundu B., Rajkhowa R., Kundu S.C., Wang X. (2013). Silk fibroin biomaterials for tissue regenerations. Adv. Drug Deliv. Rev..

[7] Liu L., Cai R., Wang Y., Tao G., Ai L., Wang P., Yang M., Zuo H., Zhao P., Shen H. (2018). Preparation and characterization of AgNPs in situ synthesis on polyelectrolyte membrane coated sericin/agar film for antimicrobial applications. Materials.

[8] Lamboni L., Gauthier M., Yang G., Wang Q. (2015). Silk sericin: A versatile material for tissue engineering and drug delivery. Biotechnol. Adv..

[9] Aramwit P., Siritientong T., Srichana T. (2012). Potential applications of silk sericin, a natural protein from textile industry by-products. Waste Manag. Res..

[10] Liu L., Cai R., Wang Y., Tao G., Ai L., Wang P., Yang M., Zuo H., Zhao P., He H. (2018). Polydopamine-Assisted Silver Nanoparticle Self-Assembly on Sericin/Agar Film for Potential Wound Dressing Application. Int. J. Mol. Sci..

[11] Nayak S., Talukdar S., Kundu S.C. (2012). Potential of 2D crosslinked sericin membranes with improved biostability for skin tissue engineering. Cell Tissue Res..

[12] He H., Cai R., Wang Y., Tao G., Guo P., Zuo H., Chen L., Liu X., Zhao P., Xia Q. (2017). Preparation and characterization of silk sericin/PVA blend film with silver nanoparticles for potential antimicrobial application. Int. J. Biol. Macromol..

[13] Kim H., Lee Y., Kim Y., Hwang Y., Hwang N. (2017). Biomimetically Reinforced Polyvinyl Alcohol-Based Hybrid Scaffolds for Cartilage Tissue Engineering. Polymers.

[14] Cai R., Tao G., He H., Song K., Zuo H., Jiang W., Wang Y. (2017). One-step synthesis of silver nanoparticles on polydopamine-coated sericin/polyvinyl alcohol composite films for potential antimicrobial applications. Molecules.

[15] Tao G., Cai R., Wang Y., Song K., Guo P., Zhao P., Zuo H., He H. (2017). Biosynthesis and characterization of AgNPs–silk/PVA film for potential packaging application. Materials.

[16] Buja A., Zampieron A., Cavalet S., Chiffi D., Sandonà P., Vinelli A., Baldovin T., Baldo V. (2012). An update review on risk factors and scales for prediction of deep sternal wound infections. Int. Wound J..

[17] Fernando S., Gunasekara T., Holton J. (2018). Antimicrobial Nanoparticles: Applications and mechanisms of action. Sri Lankan J. Infect. Dis..

[18] Zhang L., Jiang Y., Ding Y., Povey M., York D. (2007). Investigation into the antibacterial behaviour of suspensions of ZnO nanoparticles (ZnO nanofluids). J. Nanopart Res..

[19] Wang Y.W., Cao A., Jiang Y., Zhang X., Liu J.H., Liu Y., Wang H. (2014). Superior antibacterial activity of zinc oxide/graphene oxide composites originating from high zinc concentration localized around bacteria. ACS Appl. Mater. Interfaces.

[20] Lipovsky A., Nitzan Y., Gedanken A., Lubart R. (2011). Antifungal activity of ZnO nanoparticles—The role of ROS mediated cell injury. Nanotechnology.

[21] Reddy K.M., Feris K., Bell J., Wingett D.G., Hanley C., Punnoose A. (2007). Selective toxicity of zinc oxide nanoparticles to prokaryotic and eukaryotic systems. Appl. Phys. Lett..

[22] Dayakar T., Rao K.V., Bikshalu K., Rajendar V., Park S.H. (2017). Novel synthesis and structural analysis of zinc oxide nanoparticles for the non enzymatic glucose biosensor. Mater. Sci. Eng. C.

[23] Marra A., Rollo G., Cimmino S., Silvestre C. (2017). Assessment on the Effects of ZnO and Coated ZnO Particles on iPP and PLA Properties for Application in Food Packaging. Coatings.

[24] Zhang A., Neumeyer J.L., Baldessarini R.J. (2007). Recent progress in development of dopamine receptor subtype-selective agents: Potential therapeutics for neurological and psychiatric disorders. Chem. Rev..

[25] Liu Y., Ai K., Lu L. (2014). Polydopamine and its derivative materials: Synthesis and promising applications in energy, environmental, and biomedical fields. Chem. Rev..

[26] Mondin G., Wisser F.M., Leifert A., Mohamed-Noriega N., Grothe J., Dorfler S., Kaskel S. (2013). Metal deposition by electroless plating on polydopamine functionalized micro- and nanoparticles. J. Colloid Interface Sci..

[27] Kang S.M., You I., Cho W.K., Shon H.K., Lee T.G., Choi I.S., Karp J.M., Lee H. (2010). One-step modification of superhydrophobic surfaces by a mussel-inspired polymer coating. Angew. Chem. Int. Ed..

[28] Bagheri M., Rabieh S. (2013). Preparation and characterization of cellulose-ZnO nanocomposite based on ionic liquid ([C_4_ mim] Cl). Cellulose.

[29] Zhang X., Wyeth P. (2010). Using FTIR spectroscopy to detect sericin on historic silk. SCI China Chem..

[30] Kiro A., Bajpai J., Bajpai A. (2017). Designing of silk and ZnO based antibacterial and noncytotoxic bionanocomposite films and study of their mechanical and UV absorption behavior. J. Mech. Behav. Biomed. Mater..

[31] Dreyer D.R., Miller D.J., Freeman B.D., Paul D.R., Bielawski C.W. (2012). Elucidating the structure of poly (dopamine). Langmuir.

[32] Li Y., Zhang W., Niu J., Chen Y. (2012). Mechanism of photogenerated reactive oxygen species and correlation with the antibacterial properties of engineered metal-oxide nanoparticles. ACS Nano.

[33] Wang Z., Zhang Y., Zhang J., Huang L., Liu J., Li Y., Zhang G., Kundu S.C., Wang L. (2014). Exploring natural silk protein sericin for regenerative medicine: An injectable, photoluminescent, cell-adhesive 3D hydrogel. Sci. Rep..

[34] Liu J., Qi C., Tao K., Zhang J., Zhang J., Xu L., Jiang X., Zhang Y., Huang L., Li Q. (2016). Sericin/dextran injectable hydrogel as an optically trackable drug delivery system for malignant melanoma treatment. ACS Appl. Mater. Interfaces.

[35] Jalal R., Goharshadi E.K., Abareshi M., Moosavi M., Yousefi A., Nancarrow P. (2010). ZnO nanofluids: Green synthesis, characterization, and antibacterial activity. Mater. Chem. Phys..

[36] Kasemets K., Ivask A., Dubourguier H.-C., Kahru A. (2009). Toxicity of nanoparticles of ZnO, CuO and TiO_2_ to yeast Saccharomyces cerevisiae. Toxicol. In Vitro.

[37] Wu J.-H., Wang Z., Xu S.-Y. (2007). Preparation and characterization of sericin powder extracted from silk industry wastewater. Food Chem..

[38] Wang Y., Cai R., Tao G., Wang P., Zuo H., Zhao P., Umar A., He H. (2018). A novel Ag NPs/sericin/agar film with enhanced mechanical property and antibacterial capability. Molecules.

[39] Guan J., Porter D., Vollrath F. (2013). Thermally induced changes in dynamic mechanical properties of native silks. Biomacromolecules.

[40] Pal S., Tak Y.K., Song J.M. (2007). Does the antibacterial activity of silver nanoparticles depend on the shape of the nanoparticle? A study of the gram-negative bacterium Escherichia coli. Appl. Environ. Microbiol..

